# Activation of JNK Signaling Mediates Amyloid-ß-Dependent Cell Death

**DOI:** 10.1371/journal.pone.0024361

**Published:** 2011-09-14

**Authors:** Meghana Tare, Rohan M. Modi, Jaison J. Nainaparampil, Oorvashi Roy Puli, Shimpi Bedi, Pedro Fernandez-Funez, Madhuri Kango-Singh, Amit Singh

**Affiliations:** 1 Department of Biology, University of Dayton, Dayton, Ohio, United States of America; 2 Premedical Program, University of Dayton, Dayton, Ohio, United States of America; 3 Center for Tissue Regeneration and Engineering at Dayton (TREND), University of Dayton, Dayton, Ohio, United States of America; 4 Departments of Neurology and Neuroscience, McKnight Brain Institute, University of Florida, Gainesville, Florida, United States of America; Roswell Park Cancer Institute, United States of America

## Abstract

**Background:**

Alzheimer's disease (AD) is an age related progressive neurodegenerative disorder. One of the reasons for Alzheimer's neuropathology is the generation of large aggregates of Aß42 that are toxic in nature and induce oxidative stress, aberrant signaling and many other cellular alterations that trigger neuronal cell death. However, the exact mechanisms leading to cell death are not clearly understood.

**Methodology/Principal Findings:**

We employed a *Drosophila* eye model of AD to study how Aß42 causes cell death. Misexpression of higher levels of Aß42 in the differentiating photoreceptors of fly retina rapidly induced aberrant cellular phenotypes and cell death. We found that blocking caspase-dependent cell death initially blocked cell death but did not lead to a significant rescue in the adult eye. However, blocking the levels of c-Jun NH (2)-terminal kinase (JNK) signaling pathway significantly rescued the neurodegeneration phenotype of Aß42 misexpression both in eye imaginal disc as well as the adult eye. Misexpression of Aß42 induced transcriptional upregulation of *puckered (puc)*, a downstream target and functional read out of JNK signaling. Moreover, a three-fold increase in phospho-Jun (activated Jun) protein levels was seen in Aß42 retina as compared to the wild-type retina. When we blocked both caspases and JNK signaling simultaneously in the fly retina, the rescue of the neurodegenerative phenotype is comparable to that caused by blocking JNK signaling pathway alone.

**Conclusions/Significance:**

Our data suggests that (i) accumulation of Aß42 plaques induces JNK signaling in neurons and (ii) induction of JNK contributes to Aß42 mediated cell death. Therefore, inappropriate JNK activation may indeed be relevant to the AD neuropathology, thus making JNK a key target for AD therapies.

## Introduction

Alzheimer's disease (AD) is the most prevalent neurodegenerative disease, and is characterized by a gradual loss of synapses and neurons in the hippocampus and cortex, leading to a decline in cognitive function and memory [1], [2], [3], [4], [5]. The AD brain is typically associated with two types of protein deposits, amyloid plaques that contain the amyloid-ß (Aß42) peptide and neurofibrillary tangles enriched in hyperphosphorylated forms of the microtubule associated protein Tau [6]. One of the reasons of AD is the mutation in the gene that encodes Amyloid-Precursor Protein (APP) which leads to its abnormal cleavage. The normal cleavage of APP leads to the production of 40 amino acid long amyloid-beta 40 (Aß40), whereas abnormal cleavage of APP results in a 42 amino acids long polypeptide Aß42 [2], [3], [4], [5]. Aß42 is the result of ß- and γ-cleavage of the APP, and the mutations linked to familial AD affect APP and the two enzymes with γ-secreatase activity, Presenilin 1 and 2 [2], [3], [4], [5]. The ‘amyloid hypothesis’ proposes that Aß42 initiates the pathogenic cascade in AD, including aberrant cell signaling and Tau hyperphosphorylation [4]. Thus, understanding how Aß42 induces neurotoxicity [2], [7] and cell death are key questions in AD. Soluble and insoluble Aß42 assemblies cause multiple alterations to cellular homeostasis, including mitochondria dysfunction and oxidative stress, misregulation of intracellular calcium, ER stress, and aberrant signaling through interaction with several receptors. Even though a lot has been learned by modeling AD in animal model systems like mouse [2], [3] and fruit fly [3], [8], [9], [10], [11], [12], [13], [14], the exact mechanisms mediating Aß42-dependent cell death remain elusive. A hallmark of AD neuropathology as a result of Aß42 -plaques is the death of neurons [3]. Since, several genetic pathways like caspase dependent cell death pathway, P53-dependent cell death [15], [16], and c-Jun amino-terminal (NH_2_) kinase (JNK) signaling [17], [18], [19], [20] pathways are involved in cell death mechanism, it is possible that some of these pathways may be involved in neurodegeneration observed in Aß42 plaques.

The *Drosophila* eye serves as an excellent model to study patterning, growth, and is well-suited to study cell death [3], [13], [14]. The compound eye of *Drosophila* develops from an epithelial bi-layer structure present inside the larva and referred to as the eye-antennal imaginal disc. The eye-antennal imaginal disc is a complex disc, which gives rise to an eye, antenna and head cuticle of the adult fly [21], [22]. The retinal precursor cells of the eye imaginal disc undergo differentiation to form photoreceptor neurons during the third larval instar [23], [24]. Once eye differentiation is complete, the compound eye of the adult fly is comprised of about 800 units called ommatidia, each containing eight photoreceptors and several support cells. In the pupal retina, the excessive cells other than the differentiated cells are eliminated by programmed cell death (PCD) [25]. There is no PCD during earlier stages of larval eye development. However, abnormal extracellular signaling due to inappropriate levels of morphogens may trigger cell death in the developing larval eye imaginal disc [26]. Wingless (Wg), a morphogen, is known to trigger PCD in ommatidia present at the periphery of the pupal retina [27], [28] whereas ectopic Wg expression can also induce developmental cell death earlier in the developing larval eye imaginal disc [29]. In *Drosophila*, three pro-apoptotic genes: *head involution defective (hid)*, *reaper (rpr)*, and *grim* can trigger cell death by negatively regulating Drosophila inhibitor of apoptosis (DIAP1) [30], [31], [32]. DIAPs are the members of a highly conserved class of proteins, which negatively regulate caspase activity [33], [34], [35]. In response to pro-apoptotic signals, Hid, Rpr and Grim contribute to DIAP1 degradation, leading to the activation of initiator- (Dronc/caspase 9) as well as effector- caspases (Drice/caspase 3). Activation of initiator caspase triggers the caspase-dependent cell death. The caspase dependent cell death can be blocked by higher expression levels of baculovirus protein P35 [36]. However, not all the cell death is caspase-dependent. For instance, extrinsic signals like UV-irradiation that cause DNA damage and consequently trigger P53-dependent cell death [15], [16], and c-Jun amino-terminal (NH_2_) kinase (JNK) signaling pathway can induce caspase-independent cell death [17], [37], [38].

Activation of the JNK, or stress activated kinase proteins of the mitogen-activated protein kinase (MAPK) super family [17], [20], [37] may also trigger cell death due to phosphorylation of transcription factors regulating cell death [39]. It has been proposed that activation of JNK signaling leads to induction of cell death to eliminate developmentally aberrant cells, thus ensuring tissue robustness [17], [37], [40]. In *Drosophila*, JNK signaling pathway is activated downstream of the Tumor Necrosis Factor (TNF) homologue Eiger (Egr) and its receptor Wengen (Wgn) by a conserved signaling cascade that includes Tak1 (TGF- ß-activating kinase 1); a JNK kinase kinase (JNKKK), Hemipterous (Hep; a JNK kinase), Basket (Bsk; a Jun kinase), and Jun [20], [40], [41], [42]. The functional readout for the activation of JNK signaling is the expression levels of *puckered (puc)* gene, which encodes a dual specificity phosphatase, and forms a negative feedback loop by down regulating the activity of JNK [17], [37], [43]. Ectopic activation of JNK signaling has been shown to trigger apoptosis during early eye imaginal disc development [29], [44]. Although JNK signaling mediates cell death through *rpr* and *hid*, caspase inhibition does not completely prevent JNK-dependent cell death. Thus, JNK regulates apoptosis through caspase-independent mechanisms [39].

Recent observations have linked the JNK pathway to AD, including the ability of JNK to phosphorylate Tau and APP *in vitro*, promoting the accumulation of two neurotoxic species: hyperphosphorylated Tau and Aß42 [18]. Here, we demonstrate the role of JNK signaling in Aß42 neurotoxicity using a *Drosophila* model of AD. In *Drosophila*, misexpression of Aß42 in neurons of the brain resulted in decline in locomotor function, age dependent learning defects, progressive loss of neurons and reduced lifespan [3], [10], [14]. Here we demonstrate that Aß42 induces aberrant cellular morphology and increased cell death in the developing retina in late third instar eye imaginal disc. We also found that JNK signaling is activated in neurons where Aß42 is misexpressed, suggesting a role for JNK in Aß42-mediated cell death. In fact, activation of JNK signaling exacerbated Aß42 neurotoxicity, whereas downregulation of the JNK pathway prevented cell death and rescued eye size and organization. Furthermore, suppression of both JNK signaling and caspase-dependent cell death led to a suppression of Aß42 neurotoxicity in the eye, which is relatively comparable to the rescue caused by blocking JNK signaling thereby suggesting that JNK signaling mediated cell death plays an important role in AD neuropathology.

## Results

### Aß42 induces early cellular phenotypes in the developing eye disc

**Figure 1:**
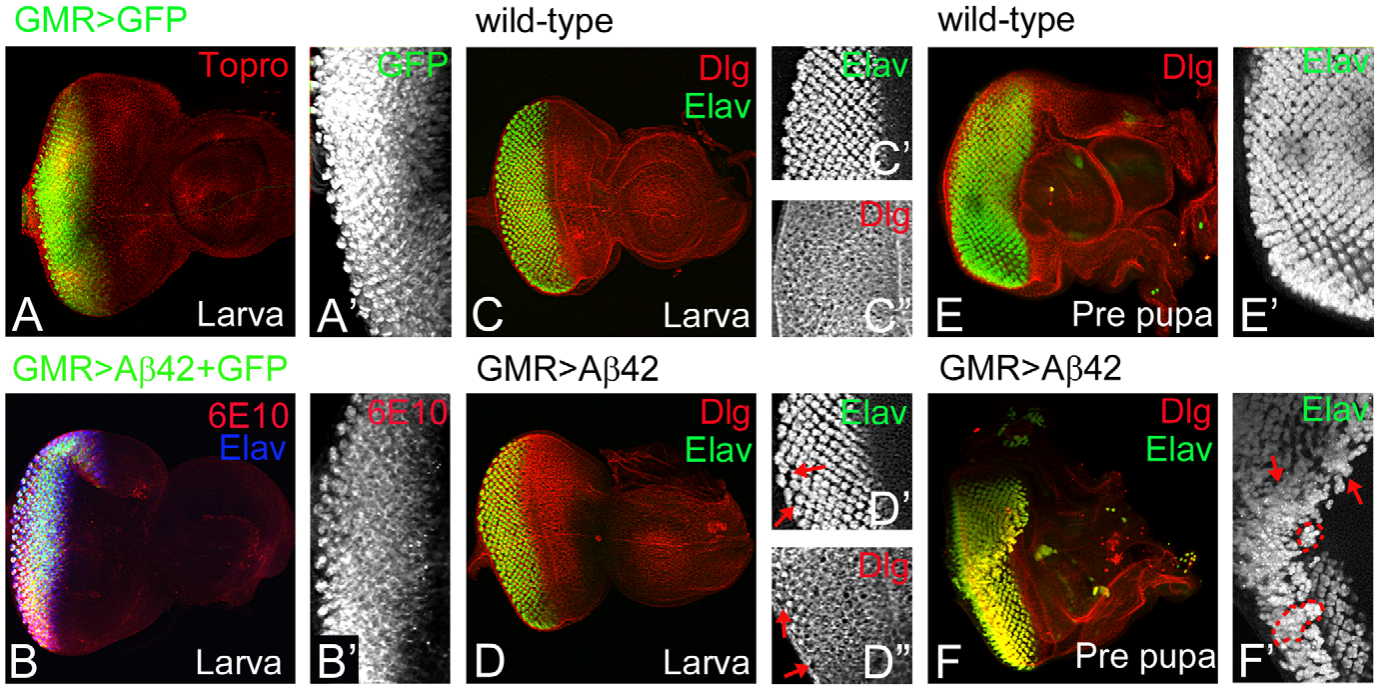
Misexpression of amyloid-beta 42 (Aß42) induces cellular phenotypes in the differentiating retinal neurons. (A, A′) GMR-Gal4 drives expression of GFP reporter (GMR>GFP) in the differentiating photoreceptor neurons in the third instar eye imaginal disc. (A′) Magnified view of GMR>GFP expression domain. (B, B′) Misexpression of Aß42 (GMR>Aß42) restricts the Aß42 expression (green channel) to the differentiating photoreceptor neurons as detected using 6E10 antibody (red channel) in eye imaginal disc. (C, E) Normal eye development in (C, C′, C″) third instar larval eye imaginal disc, (E, E′) early pupal (white pupa) retina. (D, F) Misexpression of Aß42 (GMR>Aß42) results in the induction of cellular phenotypes in the (D) developing third instar larval eye imaginal disc and (F) early pupal retina. Disc large (Dlg, red channel) marks the membrane, Elav (green channel), a proneural marker that marks the neuronal fate. (D, D′, D″) In the third instar larval stage, morphological changes are not as severe but exhibit slight “holes” (arrows) as a result of Aß42 plaque accumulation. (F) In early pupal retina, there is an increase in disarray throughout the eye field. (F′) The ommatidial clusters are clumped or fused together (arrows, outlines of ommatidial clumps marked by dotted line), (E) unlike the wild-type ommatidia.

Aß42 misexpression in the *Drosophila* eye imaginal disc induces strong phenotypes, including reduced eye size, disorganized and fused ommatidia in the adult eye [14], [45]. To understand how Aß42 exerts its neurotoxicity in the eye, we followed the early events in the development of the retina upon misexpression of Aß42. For these studies, we used GMR-Gal4 driver [46]. We employed GFP reporter to study spatio-temporal expression profile of GMR-Gal4 driver (GMR>GFP). GMR>GFP drives GFP reporter expression only in the differentiating photoreceptor neurons of the developing third instar larval eye imaginal disc (Figure 1A, A′). The misexpression of Aß42 in the larval eye imaginal discs, detected by 6E10 antibody [47], corresponds to the domain comprising of the differentiating photoreceptors, as indicated by Elav accumulation (Figure 1B, B′). The photoreceptors differentiation occurs in the third instar larval eye imaginal disc (Figure 1C, C′). The high organization of the developing eye can be appreciated by looking at the cell outlines, as indicated by the basal lamina marker Disc large (Dlg) (Figure 1C, C″) [48]. Only a few hours after Aß42 expression starts, the eye territory of the third instar eye imaginal disc exhibits subtle phenotypes. The distribution of the photoreceptors is not normal (Figure 1D and D′), the arrangement of basal membranes as shown by Dlg expression [48] indicates improper spacing (Figure 1D, D″, arrow) of the differentiating photoreceptors and mild fusion of the ommatidial clusters. Since these phenotypes worsen with aging, these abnormal photoreceptor cells are possibly basally extruded later and lost from the disc lamina.

During metamorphosis of larva to a early white pupa (prepupa), the larval eye imaginal disc with differentiated photoreceptor cells undergoes transition to a prepupal retina. Although other cell types along with the photoreceptor neurons are recruited in the prepupal retina the overall morphology still resembles an eye imaginal disc (Figure 1E, E′). A few hours after Aß42 misexpression, the neurodegenerative phenotypes of the prepupal retina get stronger (Figure 1F). In comparison to the precise arrangement of photoreceptor neurons in wild-type early pupal retina (Figure 1E, E′), the prepupal retina with Aß42 misexpression exhibits mild to stronger fusion of the ommatidial clusters (Figure 1F, F′, arrow) resulting in clumps of photoreceptor neurons and loss of photoreceptors in the ommatidium (Figure 1F, F′, arrow, dotted outline marks the ommatidial clumps).

### Analysis of Aß42 misexpression phenotypes during development

**Figure 2:**
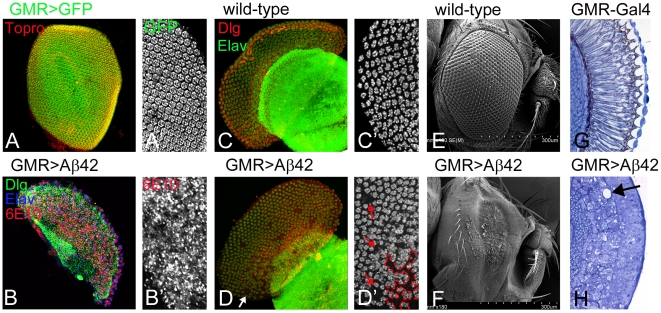
Aß42 accumulation in the developing pupal retina and adult eye results in neurodegeneration. (A, C) represent normal eye development in (A) late pupal retina, and (C) the adult compound eye. Dlg (red channel) marks the membrane; 6E10 (blue channel) marks the Aß42, and Elav (green channel), a proneural marker that marks the neuronal fate. (B, D, F) Misexpression of Aß42 in the differentiating neurons of eye using a GMR-Gal4 construct (GMR>Aß42) results in onset of neurodegeneration. (C, C′) Unlike the highly organized ommatidia of wild-type pupal retina, (D, D′) the late pupal retina shows significant neuronal cell death in the eye field. By this stage, the accumulation of Aß42 plaques has resulted in distinct holes in the eye field (marked by an arrow, outline of “hole” in pupal retina marked by red dotted line). (C) Wild type adult eye with uniform arrangement of 800 unit eyes. (I) The adult eye field of GMR>Aß42 is significantly diminished and there is complete fusion of the ommatidia. (G, H) In comparison to the adult eye section of (G) wild type fly eye, (H) the GMR>Aß42 exhibits highly disorganized morphology of photoreceptors. Furthermore the retinas of GMR>Aß42 are vacuolated. This illustrates how the neurodegenerative phenotype progressively worsens as the age and dose increase over the course of fly development.

We followed our investigation of the neurodegenerative phenotype of Aß42 misexpression in the pupal retina and the adult eye (Figure 2). The pupal retina is a highly organized structure comprising of mature ommatidia (Figure 2A, C). GMR-Gal4 drives expression of GFP reporter (Figure 2A, A′) and Aß42 in all the cell types of the pupal retina (Figure 2 B, B′). The wild-type pupal retina shows a normal array of ommatidial clusters (Figure 2C, C′) that are arranged in a hexagonal lattice whereas the pupal retina with Aß42 misexpression exhibits progressively severe phenotypes as compared to that seen in the early pupal retina (Figure 1F, F′). In GMR>Aß42 pupal retina, ommatidia do not retain the regular spacing due to multiple fusions, more of the ommatidial clusters are extruded and lost from the disc causing holes in the retina (Figure 2D, D′, holes marked by red dotted line and arrow). The GMR>Aß42 mature retina (Figure 2F) is smaller than the wild-type control (Figure 2E), suggesting that the retina undergoes considerable cell loss during prepupal stages. This small pupal retina results in a small adult eye with glazed appearance and fusion of ommatidia (Figure 2F) as compared to the wild type adult eye (Figure 2E). In the adult eye with Aß42 misexpression (GMR>Aß42), a range of neurodegenerative phenotypes were observed. We found that (∼55%) percent of the flies showed mild fusion of the ommatidium with reduced eye size (data not shown) where as the stronger phenotypes resulted in glazed appearance (∼40%) of the adult eye along with the near complete fusion of the ommatidia of the compound eye (Figure 2F). A vertical section of the wild-type adult eye shows the separation of the lenses and the depth of the photoreceptors that constitute the retina (Figure 2G). However, the adult eye sections of GMR>Aß42 indicate that the retina is very thin, has poorly differentiated photoreceptors, the lenses are fused with the underlying retina and has a vacuolated morphology (Figure 2H, arrow). Overall, these studies indicate that Aß42 induces an early pathology in photoreceptors that continues to deteriorate as the retina ages and differentiates, leading to a small and very disorganized adult eye. The gaps in early pupal retina and the small adult eye suggest that cell death may play a prominent role in Aß42 neurotoxicity in the eye. It is important to investigate how early these cells (GMR>Aß42) begin to die after Aß42 misexpression.

### Misexpression of Aß42 induces cell death in the developing eye

**Figure 3:**
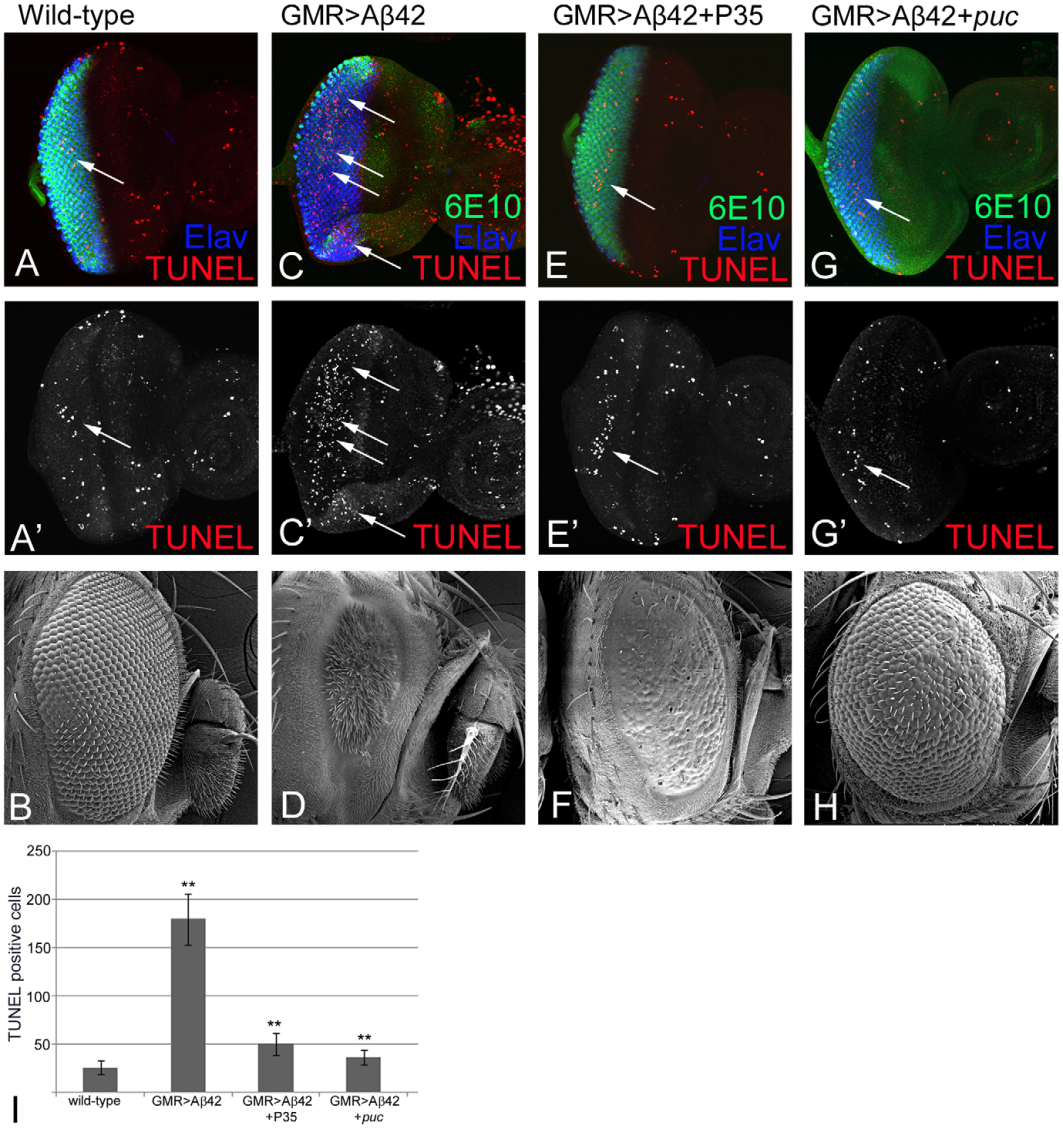
Misexpression of Aß42 triggers cell death in the differentiating neurons. (A, A′) Wild-type third instar larval eye imaginal disc displaying randomly distributed TUNEL positive dying cells (A, A″) shown in red channel (arrow). TUNEL staining marks the fragmented DNA within the nuclei of dying cells [29], [32], [61]. (B) Wild-type adult eye. (C, C′) Misexpression of Aß42 (GMR>Aß42) in differentiating neurons of the eye show elevated levels of TUNEL positive cells (C′ arrows). The increased frequency of cell death in neurons can be directly correlated to the misexpression of the Aß42 peptide. Note that misexpression of Aß42 does not affect the differentiation process as the distribution of Elav positive cells is the same in both control and Aß42 third instar eye imaginal discs. (D) GMR>Aß42 results in a strong neurodegenerative phenotype in adult eye. Baculovirus P35 has been shown to block the caspase dependent cell death [36]. (E, E′, F) Misexpression of P35 along with Aß42 in differentiating neurons (GMR>Aß42+P35) shows significant reduction of dying cells based on number of TUNEL positive cells (red channel) in the larval eye field. However, this rescue is not as strong in (F) adult eye phenotype. (E′) Note that the eye field displays reduced number of TUNEL positive cells (arrow) compared to GMR>Aß42 eye field (C′). It is important to note that Aß42 peptide production is not affected. Elav marks the photoreceptor fate (C′″). Puckered (Puc), a dual phosphatase, is downstream target of JNK signaling pathway and forms a feedback loop to negatively regulate the pathway [20]. (G, G′, H) Misexpression of *puc* along with Aß42 in the differentiating neurons (GMR>Aß42+*puc*) results in significant suppression of cell death as evident from reduced number of TUNEL positive cells in the third instar larval eye imaginal disc as well as in the (H) adult eye. Note that there is a significant rescue of (D) GMR>Aß42 adult eye phenotype in (H) GMR>Aß42+*puc* background. These results suggest that JNK signaling might be responsible for neurodegeneration seen in amyloid plaque mediated cell death. (I) Quantification of the number of dying cells in eye imaginal discs based on TUNEL staining in wild-type (served as control), GMR>Aß42, GMR>Aß42+P35 and GMR>Aß42+*puc*. Note that blocking JNK signaling (GMR>Aß42+*puc*) exhibit strong rescue of the neurodegenerative phenotype of GMR>Aß42 and GMR>Aß42+P35. This rescue is significant (**) as seen by calculation of P-values based on one-tailed *t*-test using Microsoft Excel 2007.

We performed TUNEL staining to investigate the timing of onset of cell death due to misexpression of Aß42 (GMR>Aß42) in early third instar larval eye imaginal disc. It is known that a few random cells undergo cell death in the wild-type third instar larval eye imaginal disc (Figure 3A, A′) that does not affect the final morphology of the adult compound eye (Figure 3B). We found that TUNEL positive cells, which mark the nuclei of the dying cells, begin to appear as early as late third instar eye imaginal disc (Figure 3C, C′). The number of dying cells in the GMR>Aß42 larval eye imaginal disc was significantly higher compared to the wild-type eye imaginal disc (Figure 3I). In comparison to the wild-type adult eye (Figure 3B) the Aß42 misexpression (GMR>Aß42) leads to a strong neurodegenerative phenotype of near complete loss of the adult eye (Figure 3D). In order to test whether cell death is due to induction of the intrinsic caspase dependent cell death pathway, we misexpressed baculovirus P35 along with Aß42 (GMR>Aß42+P35), and found that it resulted in partial rescue of cell death in the third instar larval eye imaginal disc. The GMR>Aß42+P35 eye imaginal disc exhibit a significant reduction in number of dying cells (Figure 3E, E′, 3I) and develop into adult flies that show subtle rescue of the adult eye field as the neurodegenerative phenotype was still present (Figure 3F). Thus, even though blocking caspase dependent cell death showed significant rescue in the larval eye imaginal disc, the adult eye showed a relatively stronger neurodegenerative phenotype suggesting that the protective role of blocking caspase dependent cell death in GMR>Aß42 is restricted to the early larval stages of eye development. Since blocking the caspases did not completely rescue the small and disorganized adult eye therefore, we tested the role of JNK pathway, a caspase-independent cell death pathway, in Aß42 neurotoxicity. To inhibit the JNK pathway, we misexpressed Puckered (Puc), a dual phosphatase that negatively regulates JNK [20], [29]. Misexpression of *puc* in GMR>Aß42 background (GMR>Aß42+*puc*) showed a significant rescue of the cell death in the eye imaginal disc (Figure 3G, G′) that resulted in a strong rescue of neurodegenerative phenotype in the adult eye (Figure 3H). Although the adult eyes have slightly disorganized ommatidia (Figure 3H, I), the extent of rescue was significantly higher than the GMR>Aß42+P35 adult eyes (Figure 3F, I). Our results suggest that although both caspase dependent as well as caspase-independent cell death through activation of JNK signaling pathway play an important role in Aß42 neurotoxicity in the *Drosophila* eye, the effects of JNK signaling was more prominent.

### Aβ42 activates JNK signaling in the eye

**Figure 4:**
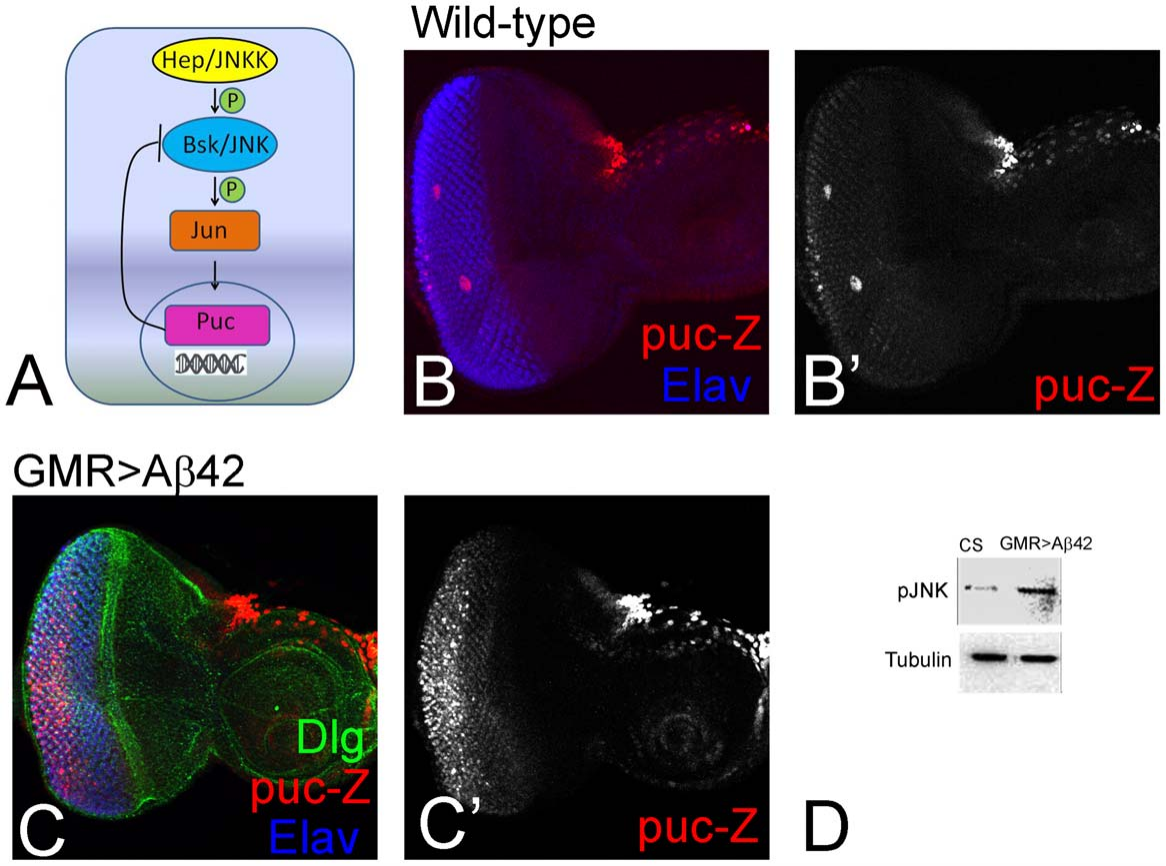
JNK signaling is activated upon misexpression of Aß42 in the eye. (A) Schematic representation of hierarchy of Jun-kinase signaling pathway members. (B, B′) Wild-type expression of *puc* in the developing third instar larval eye imaginal disc using lacZ reporter where reporter (red channel) is restricted only to the developing photoreceptors in the eye imaginal disc proper and in the peripodial membrane cells on the margin of the antennal disc [43]. (C, C′) GMR>Aß42 eye imaginal disc exhibits ectopic upregulation of *puc*-lacZ reporter. (C′) Split channel showing ectopic *puc*-lacZ expression in the photoreceptor neurons of the eye imaginal disc. (D) Activation of JNK signaling in GMR>Aß42 was detected by checking phospho-Jun levels. Levels of JNK signaling pathway increases three fold in GMR>Aß42 as compared to the wild-type eye imaginal disc.

We tested if JNK signaling pathway is activated upon accumulation of Aß42 in the eye. We analyzed the expression of *puc*, a downstream target of JNK signaling pathway (Figure 4A). Since *puc* gene is a transcriptional target of JNK signaling, the expression of *puc-lacZ* reporter serves as a functional read-out of JNK activity [43]. In the control eye imaginal disc, weak expression of *puc* enhancer trap line is detectable in photoreceptor precursors (Figure 4B, B′). However, in GMR>Aß42 eye imaginal disc, we observed strong induction of *puc*-lacZ expression (Figure 4C), especially in the most posterior domain that has expressed Aß42 longer (Figure 3C, C′). This data suggests that JNK signaling is activated in GMR>Aß42 eye imaginal disc. To confirm these results, we quantified the amount of phospho-Jun (p-Jun) present in GMR>Aß42 eye imaginal disc cells. Jun kinase (JNK) is known to encode an enzyme that can phosphorylate N-terminal of its substrate Jun [49]. The phospho-Jun quantification can provide the activation status of JNK signaling pathway. We found that in GMR-Gal4>Aß42 eye imaginal disc cells, the p-Jun levels are three times higher than the wild-type eye imaginal disc (Figure 4D). Together, this data suggests that JNK signaling is rapidly activated by Aß42 in the eye imaginal disc.

### Aß42 mediated neurodegeneration in the eye is due to activation of JNK signaling

**Figure 5:**
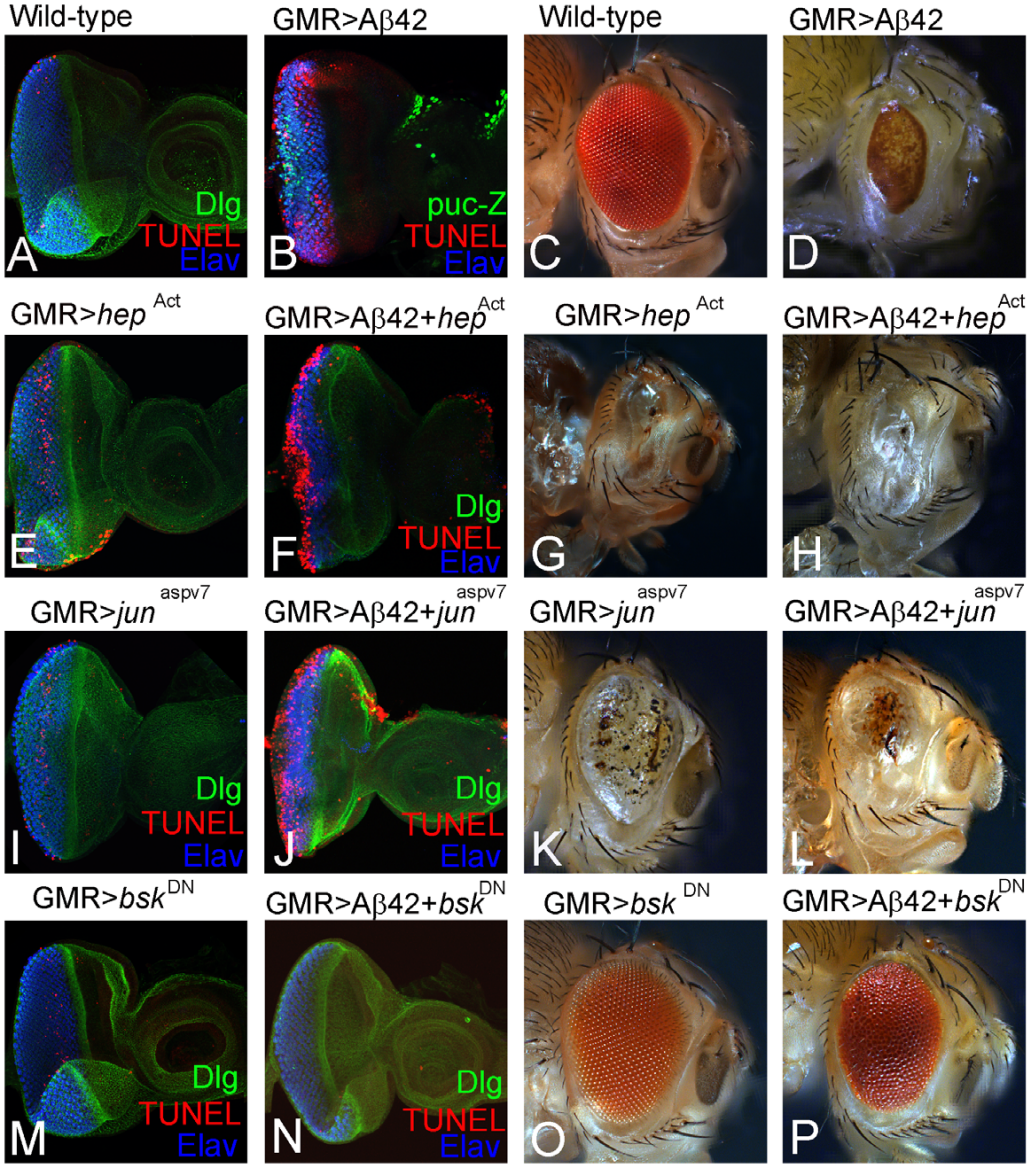
Ectopic upregulation of JNK signaling induces cell death in the GMR>Aß42 eye imaginal disc. (A) Wild-type eye imaginal disc showing cell death in random cells, which serve as controls. (B) GMR>Aß42 eye imaginal disc showing ectopic upregulation of *puc* lacZ in a large number of dying retinal cells as evident from TUNEL positive staining. In comparison to the (C) wild-type adult eye, (D) GMR>Aß42 adult eye are highly reduced due to neurodegeneration. (E–L) Increasing level of JNK signaling in GMR>Aß42 by misexpressing (F, H) activated *hemipterous* (GMR>Aß42+*hep^Act^*) and (J, L) activated *Djun* (GMR>Aß42+*jun^aspv7^*) results in (F, J) dramatic increase in dying cell population in the eye imaginal disc, leading to a (H, L) “no-eye” phenotype in the adult fly. Increased levels of (E, G) activated *hemipterous* (GMR>*hep^Act^*), (I, K) activated *Djun* (GMR>*jun^aspv7^*) served as controls and result in some dying cells in the (E, I) eye imaginal disc and (G, K) a small adult eye. However, reducing level of JNK signaling in GMR>Aß42 background by misexpressing (N, P) Dominant negative *basket* (GMR>Aß42+*bsk^DN^*) results in significant reduction to near complete absence of dying cell population (N) in the eye imaginal disc, leading to a (P) strong rescue of the adult eye phenotype as compared to GMR>Aß42 adult eye. (M, O) Increased levels of Dominant negative *basket* (GMR>*bsk^DN^*) in (M) eye imaginal disc and (O) adult eye served as controls. Note that increased levels of dominant negative *basket* alone (GMR>*bsk^DN^*) does not affect the size of eye imaginal disc and the adult eye.

We investigated the role of JNK signaling in Aß42 misexpression mediated neurotoxicity by modulating the activity of components of the JNK pathway. We found that in GMR>Aß42 background, the strong induction of *puc-lacZ* reporter in the eye imaginal disc is accompanied by dramatic increase in frequency of dying cells (TUNEL positive cells; Figure 5B) as compared to the wild-type eye (Figure 5A). Furthermore, Aß42 misexpression (GMR>Aß42) result in a strong neurodegenerative phenotype in the adult eye (Figure 5D) as compared to the wild-type adult eye (Figure 5C). Thus, if JNK signaling is involved in neurodegeneration in GMR>Aß42 background, then reducing JNK signaling levels would rescue the phenotype whereas increasing the levels of JNK signaling will have converse effect. We used several components of JNK signaling pathway (Figure 4A) to address our hypothesis and analyzed the eye phenotypes at the eye imaginal disc levels as well as the adult eye.

To activate JNK signaling, we expressed constitutively active *hemipterous* (*hep^Act^*) and *Djun* (*jun^aspv7^*). We found that misexpression of constitutively active hemipterous, GMR>Aß42+*hep^Act^* (Figure 5F) or constitutively active *Djun*, GMR>Aß42+*jun^aspv7^* (Figure 5J) enhances the frequency of TUNEL positive (dying) cells in the eye imaginal disc in comparison to their respective controls *viz.*, GMR>*hep^Act^* (Figure 5E) and GMR>*jun^aspv7^* (Figure 5I). Similar phenotypes were observed in the adult eyes of GMR>Aß42+*hep^Act^* (Figure 5H) and GMR>Aß42+*jun^aspv7^* (Figure 5L). Not surprisingly, GMR>*hep^Act^* (Figure 5G) and GMR>*jun^aspv7^* (Figure 5K) alone induce the aberrant development of the adult eye field, due to the increase in cell death. However, the phenotypes of only activation of JNK signaling pathway in eye as seen in controls are much weaker (Figure 5E, I, G, K) then when JNK signaling pathway is activated in GMR>Aß42 background (Figure 5 F, H, J, L). Thus, activation of JNK signaling significantly increases the induction of cell death by Aß42, supporting the potential involvement of JNK in Aß42 neurotoxicity.

We further tested this hypothesis by analyzing the effect of reducing the levels of JNK signaling on GMR>Aß42 neurodegenerative phenotype by using a dominant negative Basket allele (bsk^DN^). GMR>bsk^DN^, which serve as a control, does not affect developmental cell death in the eye imaginal disc (Figure 5M) and the adult eye (Figure 5O). However, misexpression of *bsk^DN^* along with Aß42 (GMR>Aß42+*bsk^DN^*) dramatically reduces the number of apoptotic cells in the eye imaginal disc (Figure 5N). This protective effect of *bsk^DN^* (GMR>Aß42+*bsk^DN^*) further continued during pupal and adult eye development, resulting in a significantly rescued, larger eye in the adult fly that still showed some ommatidial disorganization (Figure 5P). These results are consistent with the protective effect of Puc overexpression as shown in Figure 3. Overall, these studies demonstrate that JNK signaling regulates Aß42-induced cell death in the eye.

### JNK signaling plays major role in Aß42-plaque mediated cell death

**Figure 6:**
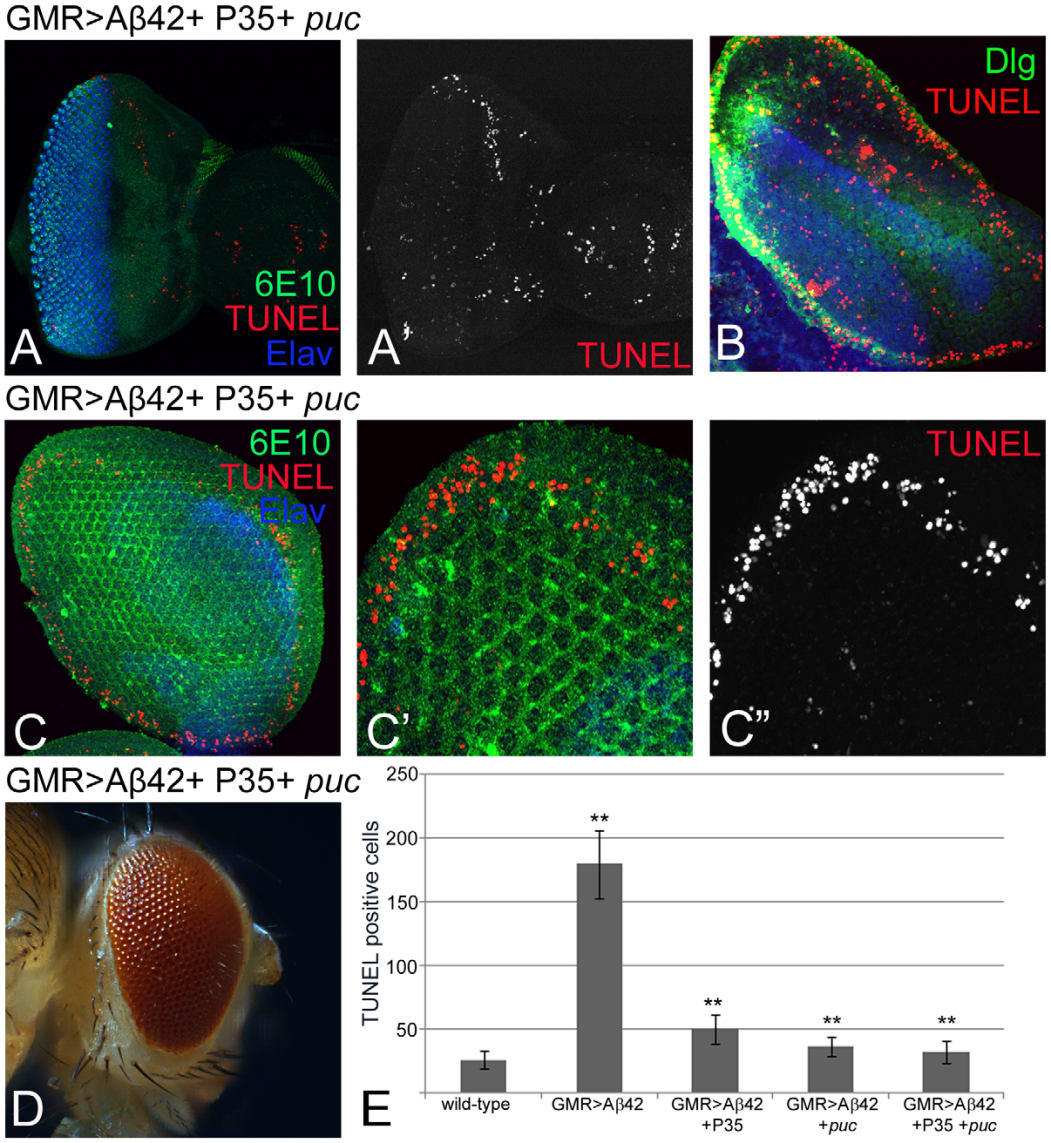
JNK signaling is responsible for cell death in GMR>Aß42 background. (A, A′) Misexpression of both P35 and *puc* along with Aß42 (GMR>Aß42+P35*+puc*) results in strong rescue of cell death as evident from (A′) dramatically reduced TUNEL positive cells. However, the rescue of the phenotype was not significantly stronger than with blocking JNK signaling pathway alone (Figure 5G′). (B) Misexpression of Aß42 (GMR>Aß42) in pupal retina showing cell death as evident from TUNEL positive cells (red channel). Blocking simultaneously both caspase-dependent cell death and caspase-independent JNK signaling mediated cell death in pupal retina (GMR>Aß42+P35*+puc*) showed a strong rescue in (C, C′, C″) pupal retina and (D) adult eye as compared to (B) GMR>Aß42 pupal retina, (Figure 3D) GMR>Aß42 adult eye. The cell death is detected by TUNEL staining (red channel), which is (C′, C″) restricted to the periphery of the pupal retina. Note that dying cells on the periphery of the pupal retina corresponds to the programmed cell death as seen in the wild-type pupal retina too [25], [28]. (E) Quantification of the number of dying cells in eye imaginal discs based on TUNEL staining in different genetic combinations. The frequency of cell death in wild-type eye imaginal disc served as a control. Note that blocking JNK signaling (GMR>Aß42+*puc*) or blocking JNK signaling along with caspase–dependent cell death (GMR>Aß42+P35+*puc*) exhibit strong rescue of the neurodegenerative phenotype of GMR>Aß42. This rescue is significant (**) as seen by calculation of P-values based on one-tailed *t*-test using Microsoft Excel 2007.

Since blocking either JNK signaling or caspase-dependent cell death alone does not result in complete rescue of the eye phenotype induced by Aß42 (Figure 3, 5), we next tested whether blocking both JNK signaling and caspase-dependent cell death pathways at the same time provided additional protection. Misexpression of both P35 and *puc* together in GMR>Aß42 background (GMR>Aß42+P35+*puc*) results in highly reduced cell death in the eye imaginal disc (Figure 6A, A′, E), even though robust levels of Aß42 are induced. However, the extent of cell death based on number of dying cells in eye imaginal disc was not significantly different from GMR>Aß42+*puc* (Figure 3E, 6E) and GMR>Aß42+P35 (Figure 3G, 6E). The pupal retina of GMR>Aß42+P35+*puc* also showed similar phenotype of highly reduced cell death (Figure 6C) as compared to GMR>Aß42 pupal retina which show significant number of TUNEL positive dying cells (Figure 6B). At this time the pupal retina shows very little cell death, which is actually restricted only to the periphery of the retina where Wg signaling eliminates the extra retinal precursor cells by PCD [28] that limits the eye/head capsule boundary (Figure 6C″). Blocking both cell death pathways (GMR>Aß42+P35+*puc*) showed a significant rescue in the adult eye (Figure 6D). However, the rescue was comparable to the one seen by blocking JNK signaling pathway alone (Figure 3H, 5M). Thus, overall the rescue by blocking JNK signaling alone (GMR>Aß42+Bsk^DN^; Figure 5K, M,) was comparable to the one seen by blocking both P35+JNK signaling (GMR>Aß42+P35+*puc*; Figure 6D). Thus, our results strongly suggest that JNK signaling pathway is activated during Aß42-plaque mediated neurodegeneration in the fly retina.

## Discussion

One of the characteristic features of neurodegenerative disorders like AD and Parkinson disease (PD) is the late onset of neuropathology due to aberrant cellular homeostasis probably due to misregulation of several signaling pathways involved in growth, patterning and survival [2], [4], [5], [50]. Thus, it is apparent that these neurodegenerative disorders are not due to a single gene mutation but a cumulative outcome of impairment of a large spectrum of signaling pathways. Therefore, in order to understand the complexity of the human disorders and to develop therapeutic approaches, it is important to discern the role of various signaling pathways in the neuropathology caused by Aß42-plaques. This evident complexity is one of the reasons why neurodegenerative diseases are so difficult to understand and treat. Our goal here was to tease out the role of the cell death pathways in Aß42 neurotoxicity. It has been known for some time that high levels of Aß42 result in small and disorganized phenotypes of eyes that contain thin retinas with poorly differentiated photoreceptors [12], [14], [45]. This small eye suggests that Aß42 induces extensive cell death in the developing eye. To understand when the cell death occurs, we studied how the maturation of photorecepotors is affected by the presence of Aß42.

We have employed the highly versatile model of *Drosophila* eye to understand the role of signaling pathways involved in cell death in Aß42-plaque mediated neuropathology [3], [10], [11], [14]. Since the eye is dispensible for the survival of fly, the transgenic *Drosophila* eye model is ideal for these studies as we can assay the effects throughout eye development without killing the fly. Our data suggest that neurodegeneration in the fly retina can be triggered as early as third instar eye imaginal disc using GMR-Gal4 driver mediated misexpression of Aß42 (GMR>Aß42; Figure 1, 3), which is only a few hours after Aß42 expression starts in the developing eye field. We also found that even though cell death is induced as early as the third instar eye imaginal disc, the morphology of the developing eye field does not dramatically differ between the wild type eye versus the GMR>Aß42. At this time the toxicity of Aß42 is only apparent at the level of cell membranes, which shows minor effects on cell arrangement (Figure 1D, D″). However, the number of the dying cells shows dramatic increase in GMR>Aß42 eye imaginal disc as compared to the wild-type eye imaginal disc (Figure 1, 3). Thus, genetic programming that triggers the onset of Aß42-plaque mediated neurodegeneration is activated soon after the onset of misexpression of Aß42 in the developing retina. Therefore, the experiments to demonstrate rescue of neurodegeneration phenotype should take this time window into consideration.

The larval eye imaginal disc metamorphose into the prepupal retina, which shows clumping of photoreceptor clusters, an indication that photoreceptor specification and signaling are aberrant (Figure 1F. F′). The clumping phenotype is caused by fusion of photopreceptor neurons and results in loss of ommatidial cluster integrity. Despite these changes at the photoreceptor neurons level, the outline of the pupal retina shows subtle effects (Figure 1). In the late pupal retina, the size of the retina begins to reduce as the severity of the phenotypes increases at this stage. In the late pupal stage, the retina contains holes due to loss of photoreceptors. The outcome of this cellular aberrations in the eye leads to a small adult eye with glazed appearance and fused ommatidia. Thus, extensive cell death is responsible for some of the phenotypes observed in the adult eye expressing Aß42. Not surprisingly, the neurodegenerative phenotypes exhibited by Aß42-plaque are age and dose dependent. Since the Gal4-UAS system is temperature sensitive, it serves as an excellent source to test the dose dependence [51], [52]. The cultures reared at 25°C showed less severe phenotypes as compared to the ones reared at 29°C (data not shown). Furthermore, the severity of phenotypes increased with the age (Figure 2).

The next plausible question was, which pathways mediate the extensive cell death induced by Aß42? Our idea was to test the caspase-dependent pathway since the majority of cell death is triggered by activation of caspase-dependent cell death in tissues. To demonstrate the role of caspases in Aß42-mediated cell death, we show that the misexpression of baculovirus P35 protein [36], significantly reduce the number of TUNEL-positive cells in the larval eye disc (Figure 6E). Interestingly, unlike the larval eye disc, the adult eyes did not show comparable strong rescues. It seems there is block in cell death mainly during the larval eye imaginal disc development but the adult eye exhibits a weaker rescue of GMR>Aß42 neurodegenerative phenotype. This reduction in cell death supports the possible role of caspase-mediated cell death in the small eye induced by Aß42. However, the eye of GMR>Aß42+P35 is reduced and disorganized (partial rescue), suggesting that other pathways contribute to Aß42 neurotoxicity in the eye.

### Aß42 neurotoxicity is mediated through activation of JNK signaling pathway

JNK-mediated caspase-independent cell death also plays an important role in tissue homeostasis during development. JNK signaling, a family of multifunctional signaling molecules, is activated in response to a range of cellular stress signals and is a potent inducer of cell death [20]. Consistent with this, Aß42 activates JNK signaling in the eye imaginal disc as indicated by the transcriptional regulation of *puc* and Jun phosphorylation (Figure 4). Moreover, JNK signaling upregulation increases cell death, supporting the role of JNK in Aß42 neurotoxicity (Figure 5). Conversely, blocking JNK signaling dramatically reduces cell death in larval eye imaginal disc (Figure 6E) and the resulting flies from blocking JNK signaling exhibit large and well organized eyes (Figure 5). Thus, we were able to identify the JNK signaling pathway as a major contributor to cell death observed in the Aß42 eyes. Our studies also highlight that cell death response to misexpression of Aß42-plaques is way earlier before its affect can be discernible at the morphological level. Since neurons are post-mitotic cells, they can not be replaced. Therefore, early detection of the onset of neurodegeneration is crucial. If the disease is detected later, it may only be possible to block the further loss of healthy neurons. However, the neurons lost prior to block of cell death will not be replaced. It is possible that JNK signaling activation may serve as an early bio-marker for Aß42 plaque mediated neuropathology. Thus, members of JNK signaling pathway can serve as excellent biomarkers or targets for the therapeutic approaches.

We found that blocking JNK signaling significantly rescued the neurodegenerative phenotypes but the eyes still show subtle signs of Aß42 in the disorganization of the lattice. Therefore, we blocked both caspase dependent cell death and JNK signaling in fly retina misexpressing Aß42. Blocking both caspase and JNK pathways simultaneously produced the protection against Aß42, suggesting that Aß42 induces cell death by several mechanisms. Our results suggest that blocking multiple pathways may result in significant protection against Aß42 neurotoxicity, an important consideration for potential AD therapies.

### JNK signaling pathway plays role in cell survival

JNK signaling pathway has been known to be involved in different processes of ageing and development, including tissue homeostasis, cell proliferation, cell survival and innate immune response. Interestingly, evidence collected in several models of AD supports the involvement of JNK signaling in AD. Consistent with our observations, Aß42 induces JNK activation in primary cultures of rat cortical neurons [53]. Also, the kinase activity of JNK phosphorylates Tau *in vitro*, thus contributing to the production of hyperphosphorylated Tau, one of the key toxic molecules in AD [54]. Moreover, inhibition of JNK with peptides prevented cell loss in an Tg2576; PS1^M146L^ brain slice model [18]. Additionally, it has been shown that the neuroprotective effect of the diabetes drug rosiglitazone inhibits JNK and results in reduced Tau phosphorylation in rats and mice [55]. Our results support these findings in mammalian models of AD, and provide the first evidence that direct manipulation of JNK activity modulates Aß42 neurotoxicity *in vivo*. Despite this evidence, JNK is currently not a major pathway in AD research. Our results, together with the published literature, suggest that more attention should be paid to the role of JNK in AD pathogenesis and its potential as a therapeutic target and biomarker. In fact, the protective activity of JNK may not be limited to AD, as JNK inhibition may show beneficial effects in other diseases, including PD, stroke and others [49].

## Materials and Methods

### Stocks

Fly stocks used in this study are described in Flybase (http:// flybase.bio.indiana.edu). Stocks used include: GMR-Gal4 UAS-Aß42, and *puc ^E69^*, a *lacZ* reporter which expresses under the control of *puc* endogenous regulatory elements acts as a functional readout of JNK signaling pathway [43] .

We have used Gal4/UAS system for targeted misexpression studies [56]. All Gal4/UAS crosses were done at 18°C, 25°C and 29°C, unless specified, to sample different induction levels. All the experiments were conducted using the Glass Multimer Reporter driver line (GMR-Gal4), which directs expression of transgenes in the differentiating retinal precursor cells of the developing eye [46]. The responder strains were: UAS- Aß42 [14], [45] , UAS*-bsk^DN^* [37], UAS-*DJun^aspv7^* [57], UAS-*puc* [43], UAS*-p35* [36], P{UAS-GFP.S65T/T10} [58].

### Immunohistochemistry

Eye-antennal imaginal discs were dissected from wandering third instar larvae and stained following the standard protocol [59]. Pupal retina were dissected and fixed in 4% paraformaldehyde and stained with combinations of antibodies using the standard protocol [59], [60]. Antibodies used were mouse and rabbit anti- ß-galactosidase (1∶200) (Cappel); rat anti-Elav (1∶100); mouse anti-Wg (1∶50) (Developmental Studies Hybridoma Bank); mouse anti-6E10 (1∶100), {[47], Cell Science.com}, rabbit anti-Dlg (a gift from K. Cho). Secondary antibodies (Jackson Laboratories) were goat anti-rat IgG conjugated with Cy5 (1∶200), donkey anti-rabbit IgG conjugated to Cy3 (1∶250), donkey anti-rabbit IgG conjugated to FITC, donkey anti-mouse IgG conjugated to Cy3 (1∶200). Immunofluorescent images were analyzed using the Olympus Fluoview 1000 Laser Scanning Confocal Microscope. All final figures were prepared using Adobe Photoshop software.

### Detection of Cell Death

Apoptosis was detected by using TUNEL assays [29], [32], [61]. TUNEL assays are used to identify cells undergoing apoptosis where the cleavage of double and singled stranded DNA is marked effectively. This protocol involves labeling DNA breakage by adding fluorescently labeled nucleotides to free 3′-OH DNA ends in a template-independent manner using Terminal deoxynucleotidyl transferase (TdT). The fluorescein labels incorporated in nucleotide polymers can be detected by fluorescence microscopy. Eye-antennal discs, after secondary-antibody staining [59], were blocked in 10% normal goat serum in phosphate buffered saline with 0.2% Triton X-100 (PBT) and labeled for TUNEL assays using a cell-death detection kit from Roche Diagnostics. The TUNEL positive cells were counted from five sets of imaginal discs and were used for the statistical analysis using Microsoft Excel 2007. The P-values were calculated using one-tailed *t*-test, and the error bars represent Standard Deviation from Mean.

### Histology

For histological analysis of retinas, epon-embedded heads of one day-old flies were sectioned at 1 µm and stained with toluidine-blue as described before [62]. Sections were documented in a Nikon 80i microscope with a Zeiss Axiocam digital camera and AxioVision software.

### Western Blot

Protein samples were prepared from eye-antennal imaginal discs from third instar wild type and GMR>Aß42 larvae in PBS and then subjected to boiling in Lamaelli's sample buffer containing SDS- ß mercaptoethanol for 10 minutes. Samples were resolved on a 10% gel, and transferred on to nitrocellulose membrane. The blot was washed with 1× TBST (pH 7.5) for 10 min each (X3), incubated in 5% w/v BSA in 1× TBST (pH 7.5) overnight. The blot was recovered from blocking solution the following day, and incubated in diluted 1∶1000 Phospho-SAPK/JNK (Cell Signaling Thr183/Tyr185) (81E11) Rabbit antibody diluted in 5% w/v BSA in 1× TBST at 4°C with gentle shaking overnight. Signal was detected using horseradish peroxidase-conjugated goat anti-rabbit IgG using super signal chemiluminescence substrate (Pierce). Images were captured using the BioSpectrum® 500 Imaging System.

### Scanning Electron Microscopy (SEM)

The flies were prepared for scanning electron microscopy through a series of increasing concentrations of acetone [63]. Dehydrated flies were then incubated in 1∶1 acetone and HMDS (Hexa Methyl Di Silazane, Electron Microscopy Sciences) for 24 hrs followed by incubation in 100% HMDS. The flies were allowed to air dry in HMDS in the hood. Dehydrated flies were mounted on Electron microscopy stubs. Flies were coated with gold using a Denton vacuum sputter coater and analyzed using a Hitachi S-4800 High Resolution Scanning Electron Microscope (HRSEM).
